# Delays in Cancer Diagnostic Testing at a Quick Referral Unit in Spain during COVID-19

**DOI:** 10.3390/diagnostics11112096

**Published:** 2021-11-12

**Authors:** Xavier Bosch, Manuel Torres, Pedro Moreno, Alfonso López-Soto

**Affiliations:** Department of Internal Medicine, Hospital Clínic, University of Barcelona, 08036 Barcelona, Spain; MTORRESE@clinic.cat (M.T.); PJMORENO@clinic.cat (P.M.); ALOPEZ@clinic.cat (A.L.-S.)

**Keywords:** COVID-19, cancer diagnosis, quick diagnosis units, suspected cancer, primary care

## Abstract

Although COVID-19 has had dire consequences on diagnosis of cancer, little data assessing its impact on the whole range of diagnostic activity relevant to cancer are available. We examined trends in the provision of full diagnostic tests for consecutive patients with suspected cancer referred to an academic hospital-based Quick Diagnosis Unit from January 2019 to December 2020. As weekly volumes declined, waiting times for endoscopic, imaging and biopsy/cytology procedures increased steeply during the COVID-impacted period (26 February–28 April 2020). The average weekly increase compared with the same period in 2019 was substantial for invasive procedures requiring admission (200.70%), CT scans (171.20%), GI endoscopy (161.50%), PET/CT scans (152.50%), ultrasonography (148.40%), and ambulatory biopsy/cytology procedures (111.20%). Volumes and waiting times to other procedures showed similar trends. There was a remarkable downward trend in cancer diagnosis during the COVID-impacted period, with a 54.07% reduction compared with the same weeks in 2019. Despite a modest recovery in the following months, the decline in weekly activity and cancer rates persisted until 30 December. Providing insight into how COVID-19 changed the full spectrum of diagnostic activity for suspected cancer informs resilience-building interventions to guarantee access to fast and efficient diagnostics ahead of new threats.

## 1. Introduction

Patients with cancer are commonly diagnosed after seeking care for associated symptoms at primary care (PC) centers. Based on suspected symptoms, physical examination findings, and perhaps some initial tests, these patients may be referred to specialized healthcare to make a diagnosis and, if cancer is confirmed, consider treatment options. Alternatively, diagnosis of cancer sometimes occurs after patient presentation to the emergency department (ED), in which case a more advanced stage with poorer outcomes is expected [1,2].

People with suspected cancer in several countries are managed through specific pathways. Rolled out as alternatives to hospital admission, Quick Diagnosis Units (QDUs) were conceived in Spain around the middle of 2000s for timely diagnosis of potentially serious diseases including symptomatic organic diseases and cancers with both narrow and broad symptom signatures [3]. This model bears resemblances to the Rapid Diagnostic Centre (RDC) service being rolled out across the UK to support faster diagnosis of cancer in patients referred from PC with nonspecific but concerning symptoms that could indicate cancer [4]. Based on a similar model in Denmark [5], the RDC concept was born out of the recognition that there was no urgent diagnostic pathway for these patients in the UK. In contrast, the Spanish QDU concept was born from the realization that the country healthcare system relied largely on ‘bed-based’ inpatient care [6]. To offer care in the best setting, instead of ending up in acute hospitals by default, an innovative range of hospital-based outpatient and ambulatory care strategies were set up, including particularly QDUs [6,7]. At present, QDUs are running in most public hospitals from the northeastern regional community of Catalonia and are managed by Internal Medicine specialists. These units have proven to be effective in preventing unnecessary PC referrals to ED. Analyzing patients referred from PC to ED, then referred from ED to QDU, an article published in a family medicine journal revealed that >90% of them could have been safely referred first to QDU, without need for going to ED [8]. Furthermore, when assessing the impact of PC referrals to QDU through two periods of 2 years comparing PC doctors that had been educated about the role and evaluation criteria of the unit, there was a substantial change in the pattern of referrals over time with an increasing number of patients referred directly to the QDU rather than ED, which contributed to alleviating ED overcrowding and reduce the number of emergency admissions and hospitalization costs [9].

Studies evaluating nonspecific symptom pathways have shown diagnostic yield estimates for cancer ranging from 8% in the UK to 11% and 22% in Denmark and Sweden [5,10,11]. The conversion rate is even higher in QDUs reflecting a broader scope of referral criteria (i.e., both specific and nonspecific symptoms). Either way, patients diagnosed with cancer after symptomatic presentation to their PC provider are among those groups requiring time-critical access to medical services for urgent investigation [12]. For them, only timely diagnosis with detection at a treatable stage can make a difference in outcomes, as described in a recent systematic review showing even a 1-month delay in treatment was associated with increased mortality across three major treatment modalities for seven cancers [13]. During 2020, however, access to medical diagnostics has been severely disrupted due to the coronavirus disease 2019 (COVID-19) pandemic, with modeling studies from the UK estimating that within the next 5 years delayed diagnosis and late-stage presentations will cause >3000 additional deaths from breast, lung, esophageal and colorectal cancer [14]. The healthcare system was disrupted in Spain during the pandemic and the impact of the COVID pandemic on cancer care has not been fully investigated. Using population-based e-health registries from PC, a recent report recounted a 34% reduction in the incidence rates of cancer during the COVID-19 period March–September 2020 in Catalonia. Although the study reported the degree of change in the volume of mammograms and colonoscopies performed during this period, other diagnostic tests were not mentioned [15].

With nearly 2 million cases and 51,000 deaths by 30 December 2020, the responses to the pandemic in Spain were much the same as in other countries and included reductions in the number of patients seeking healthcare and in hospital admissions, virtual interruption of PC services with a massive shift from face-to-face to telehealth visits, and major shortfalls in medical services for non-COVID-19 patients [16,17]. A recent article in an emergency medicine journal described the management of patients suspected of having cancer referred by ED to QDU during the first half of 2020. The study revealed a significant downward trend in referral volumes and highlighted the difficulties in gaining access to medical services as the hospital switched its usual care to one slanted towards COVID-19 [18]. Besides the overshadowing effect from COVID-19 on healthcare, there was an unexpected increase in the rates of functional, mental, and behavioral health disorders, which were linked to general reactions of risk avoidance [18]. Since PC constitutes the original source for 40–50% of patients referred to the unit and 20–30% of them have an eventual diagnosis of cancer, providing insight into how COVID-19 changed diagnostic activity on this population can inform decision-making to ensure that cancer services are protected and patients coming forward with concerning symptoms have access to the best possible medical diagnostics.

The purpose of this study was to investigate the effects of COVID-19 on the full provision of key diagnostic tests in patients with symptoms suggestive of cancer referred from PC to QDU in a public academic hospital in Spain including the periods before the first outbreak, the spike and lockdown, through to the end of 2020.

## 2. Materials and Methods

### 2.1. Study Design and Setting

This was a retrospective observational study of all consecutive adult patients referred to the QDU of our Institution (Hospital Clínic of Barcelona-a tertiary university hospital) between 1 January 2019, and 30 December 2020. This Institution is the first point of contact with health services for many individual patients within the hospital and the community and provides complex clinical care to patients referred from 16 PC centers and secondary-level hospitals from the Barcelona area. Collectively, Hospital Clínic and its associated centers provide healthcare for a population of 700,000. The city was the epicenter of the COVID-19 pandemic in Catalonia. The unit is located at an ambulatory daycare center and is staffed with Internal Medicine consultants and residents, Family Medicine residents, registered nurses, and administrative workers. As a fundamental principle governing referral and evaluation, patients’ performance status is expected to be well enough to let them go to hospital for appointments and examinations, then returning home [3,9].

### 2.2. Study Population

Eligible patients were those aged >18 years who were newly referred from PC centers for investigation of suspected cancer during 2019 and 2020. Patients had to meet predefined criteria for referral [3,9], outlined in Supplementary Table S1. Exclusion criteria applied to referrals from sources other than PC, patients whose performance status (or added comorbidity) recommended management through alternative pathways, patients who were lost or died before a diagnosis was made, when data were not present in the e-health records, and patients with severe acute respiratory syndrome coronavirus 2 (SARS-CoV-2) infections. This study was performed according to institutional ethical guidelines for medical research. Board approval was obtained for this retrospective study.

### 2.3. Data Collection and Measurements

Information was extracted from the e-medical database of the hospital. To investigate changes over time in reported healthcare quality indicators of QDUs [7,19], data were retrieved on all investigative tests and procedures undertaken each 7-day time interval over 2 years. In addition to volumes of referrals, procedure volumes and waiting times (between date of request and date of procedure) were examined. Investigations and procedures included gastrointestinal (GI) endoscopy/endoscopic ultrasonography (USE), computed tomography (CT) scan, positron emission tomography integrated with CT scan (PET/CT scan), ultrasonography, invasive procedures requiring admission (CT/ultrasound-guided biopsy of abdominal organs such as liver biopsy and endoscopic retrograde cholangiopancreatography (ERCP)), and ambulatory biopsy/cytology procedures (peripheral lymph nodes/lumps and CT/ultrasound-guided procedures for non-abdominal organs/tissues). Volumes and waiting times of bronchoscopy/endobronchial ultrasonography, magnetic resonance image, bone scintigraphy, and bone marrow biopsy/aspiration were also analyzed. Waiting times of interest also included the interval between date of referral and appointment to QDU and time-to- diagnosis (from first appointment to date of diagnosis). Further metrics related to QDU activity in 2019 and 2020 were mean number of appointments per case, ratio of successive/first visits, and ratio of telehealth/total visits.

To describe the characteristics of the overall cohort, data collected included demographics (age, sex, household annual income per capita), smoking history (current, former, none), alcohol intake (excessive, normal limits, none), comorbidity score (according to Charlson’s index (0–1, 2, >3)) [20], clinical manifestations prompting referral, and diagnosis (cancer, non-malignant organic disorders, other). For patients with cancer diagnosis, we recorded whether presenting symptoms were mainly focal or nonspecific, performance status [according to Eastern Cooperative Oncology Group Performance Status scale: 0–1 (absent/minor impairment), 2 (moderate impairment), 3–4 (severe impairment)] [21], pre-referral consultations (number of consultations with the PC physician before referral to QDU) (1, 2, >3), primary tumor site, and tumor stage (I-II, III-IV). Cancers were coded according to the International Classification of Diseases for Oncology (ICD-O-3) [22]. Tumor stage was based on the TNM Classification of Malignant Tumors [23].

### 2.4. Statistical Analysis

Trends in weekly volumes and waiting times in 2019 and 2020 were first noticed by scatterplot visualization of data. We identified time cut points associated with changes in volumes in 2020. Four periods were defined: (1) stable pre-COVID, from 1–7 January to 19–25 February; (2) COVID-impacted, from 26 February–3 March to 22–28 April, around the time of maximum transmission rate of SARS-CoV-2 and first lockdown on March 14 [24]; (3) de-escalation, from 29 April–5 May to 24–30 June, characterized by a progressive lifting of restrictions and end of lockdown on 21 June; and (4) second wave, from 1–7 July to 23–30 December, with an upsurge in cases and new state of emergency including a national curfew on 25 October [25]. January/February 2020 were chosen as pre-pandemic comparator months owing to the proximity to the onset of pandemic. To confirm that these months were representative of a non-pandemic period, the main study variables were compared between January/February 2020 and January/February 2019 showing no significant differences (Supplementary Table S2).

Percent differences in 2020 metrics, stratified by procedure types, were calculated by week for pre-COVID, COVID-impacted, de-escalation and second wave periods, and compared with 2019 figures. Differences in the 2020 calendar year were compared with the same weeks in 2019 to assess the degree of change accounting for monthly or seasonal variation within the same calendar year. Changes are shown with 95% confidence intervals (CI) based on the ratio of two rates (assuming a Poisson distribution).

Quantitative variables are expressed as means (standard deviation) in case of normal distribution or medians (interquartile range) otherwise. Categorical variables are expressed as numbers (percentage). Normality of continuous data was assessed using histograms and confirmed with the Shapiro–Wilk test. For all relevant metrics, mean weekly values were calculated for pre-COVID, COVID-impacted, de-escalation, and second wave periods, stratified by procedure types. Independent-samples *t*-tests were performed comparing the mean weekly values in years 2020 and 2019 for each period. Threshold for statistical significance was established a priori at *p* < 0.05. Data that were not present in the e-health database were excluded from analysis and no imputation was performed. Statistical analyses were performed using GraphPad Prism v8 (GraphPad Software, San Diego, CA, USA).

## 3. Results

### 3.1. Study Population

**Figure 1:**
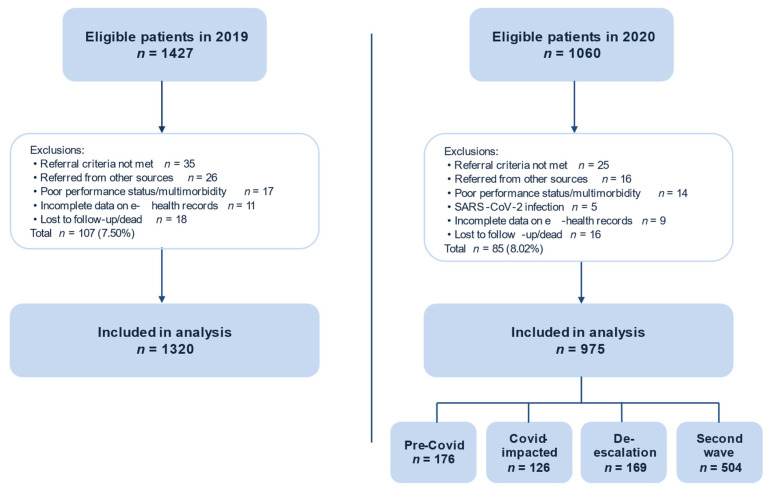
Study flow diagram. Inclusion and exclusion criteria are described in Methods. Abbreviations: SARS-CoV-2 = severe acute respiratory syndrome coronavirus 2.

Among 1427 initially eligible patients in 2019, 107 (7.50%) were excluded, leaving 1320 who were referred from 1 January to 30 December with symptoms suggestive of cancer. After excluding 85 of 1060 (8.02%) eligible patients in 2020, there were 975 who were referred from 1 January to 30 December including 176 patients in pre-COVID, 126 in COVID-impacted, 169 in de-escalation, and 504 in second wave periods (Figure 1).

### 3.2. Referral Volumes

**Figure 2:**
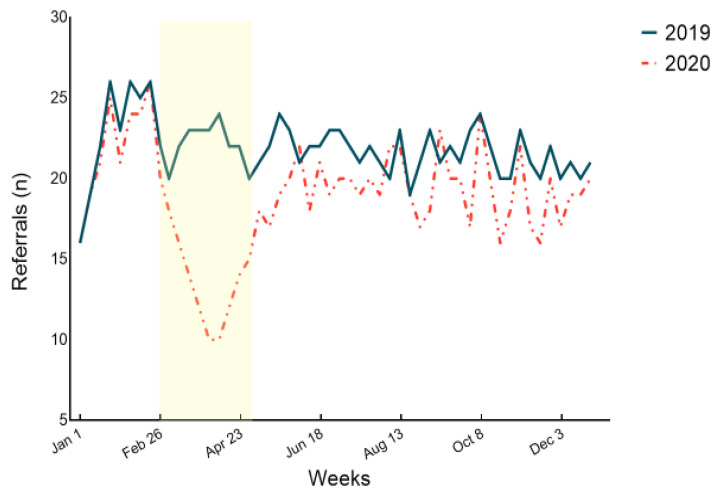
Weekly volume of referrals in 2019 and 2020. The shaded area depicts the COVID-impacted period (26 February–28 April 2020) which included the first national lockdown on 14 March. The de-escalation period went from 29 April to 30 June and the second wave period from 1 July to 30 December.

After increasing during January 2020 and 2019 (a characteristic trend following the Christmas period), referrals declined sharply during the COVID-impacted period in 2020, then increasing during de-escalation and second wave periods (Figure 2). Compared with the weekly values in the same periods in 2019, the number of referrals decreased by 36.66% (95% CI: 22.72–50.60) during COVID-impacted, 14.58% (95% CI: 7.33–21.83) during de-escalation, and 9.30% (CI 95%: 5.76–12.83) during second wave periods.

### 3.3. Characteristics of Study Population

Table 1 shows the general characteristics of patients from the COVID-impacted period and the same period in 2019. No significant differences were observed in the mean age (68.22 years in 2019 vs. 66.35 years in 2020) and gender distribution (males: 53.73% in 2019 vs. 51.59% in 2020). Patients from the 2019 period had a higher number of comorbidities than those from the 2020 period without reaching statistical significance. The frequency of symptoms prompting referral by PC clinicians in 2019 and 2020 was similar. Main presenting symptoms in the 2020 COVID-impacted period included unexplained weight loss/fatigue in 17.46% and anemia in 14.29% of patients. Fever of unknown origin, abdominal pain, and masses were also relatively common. Cancer was diagnosed in 15.87% and 21.39% of patients from the 2020 and 2019 periods, respectively. Presenting symptoms were nonspecific in about one-third of patients from both periods. Cancer patients from 2020 had significantly lower performance scores than those from 2019. Significant differences were also observed in the number of pre-referral consultations (1 or 2 in 90.00% of 2020 patients vs. 76.74% of 2019 patients; *p* = 0.0092). Nonmalignant organic disorders in patients from 2020 most included gastrointestinal (22.62%), hepatobiliary/pancreatic (11.90%), and rheumatic/autoimmune/granulomatous diseases (5.95%) without relevant differences vs. 2019 patients (Table 1).

### 3.4. Appointments and Procedure Volumes

The mean number of referrals, appointments per case and ratio of successive/first visits in each period of the study in 2020 are shown in Table 2. There was a substantial increase in the ratio of telehealth visits in the COVID-impacted period, making up 58.51% of all appointments. Table 2 displays the weekly volumes of endoscopic, imaging, invasive, and ambulatory biopsy/cytology procedures in each study period in 2020 and the percent differences compared with the same periods in 2019. As a result of the pandemic starting up, GI endoscopy/USE procedures declined from 8.44 ± 2.70 in 2019 to 4.78 ± 2.17 during the COVID-impacted period (*p* = 0.0058), representing an average reduction of 42.04% (CI 95%: 22.66–61.42). Despite a gradual recovery through de-escalation and second wave, volumes did not return to 2019 levels. In the second wave period, the total volume of GI endoscopy/USE procedures decreased by 14.24% compared with the same weeks in 2019. As shown in Table 2 and Table S3, across all modality types, volumes declined during the COVID-impacted period. In all cases, no statistical differences in mean weekly values were seen between the pre-COVID period in 2020 compared with 2019. However, when comparing volumes in each pandemic phase with the same weeks in 2019, differences and declines were remarkable across all modality types, especially in the COVID-impacted period.

### 3.5. Waiting Times

Mean times to appointments, procedures, and diagnosis in each study period are presented in Table 3. Percent changes relative to 2019 are shown for the pre-COVID period in 2020 and for each pandemic period. The same table displays how mean weekly values compared between the full pandemic period 26 February–30 December and the same 26-week period in 2019.

**Figure 3:**
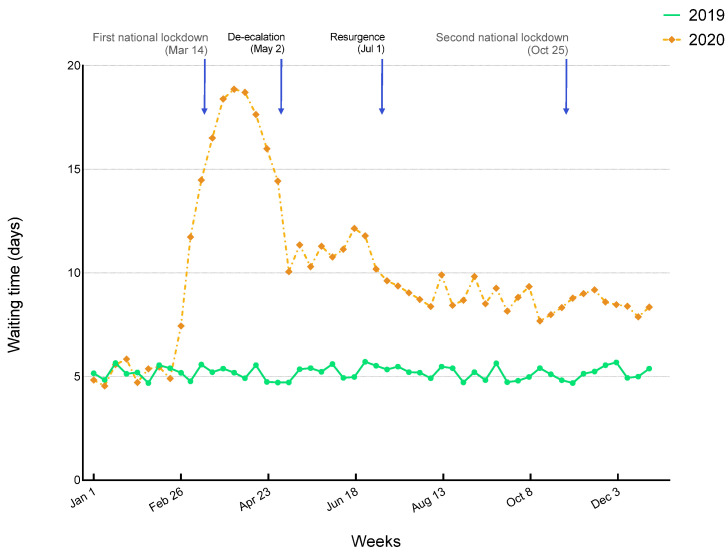
Trends in waiting times to invasive procedures by calendar week during COVID-19 pandemic.

Waiting times to QDU appointments and procedures increased steeply during the COVID-impacted period. Compared with the same periods in 2019, times to GI endoscopy/USE increased by 161.50% (95% CI: 111.60–211.40) during the COVID-impacted period and remained above non-pandemic values during the second wave. Among all procedures, the greatest increase was seen for invasive procedures requiring admission. From 5.17 ± 0.31 days in the previous year, the waiting time increased to 15.53 ± 3.81 days in the COVID-impacted period (*p* < 0.0001), which represented an increase of 200.70% (95% CI: 144.20–257.20) (Figure 3). Despite a moderate improvement during de-escalation, there was a 70.64% increase (95% CI: 65.46–75.82) during the second wave period and a 60.32% increase (95% CI: 49.83–70.81) during the last 6 weeks of the study (18 November–30 December). For all procedure types, waiting times showed similar trends (Table 3 and Table S3). Because of long waiting times, time-to-diagnosis increased substantially. In the pre-COVID periods of 2020 and 2019, time-to-diagnosis was 10.38 ± 0.59 and 10.35 ± 0.67 days, respectively (*p* = 0.9258). However, a sharply increasing trend was observed during the COVID-impacted period, with a 97.04% (95% CI: 65.15–128.90) rise compared with the same 9-week period in 2019 (20.14 ± 3.72 vs. 10.28 ± 0.71 days, respectively; *p* < 0.0001).

### 3.6. Cancer Diagnosis

Trends in numbers of cancer across each study period in 2020 are displayed in Table 4. Relative to the same periods in 2019, there was a 54.07% (95% CI: 32.45–75.70) reduc-tion during the COVID-impacted period and an average reduction of 8.06% (95% CI: −26.26–42.37) during the last 6 weeks of the study (18 November–30 December). Pancreatic, colorectal, hematological, and lung cancers were the most common malignancies in all periods. Out of 142/799 (17.77%) malignancies diagnosed throughout all pandemic periods in 2020, 18.31% were colorectal, 17.61% pancreatic, 15.49% hematological (mostly lymphomas), and 13.38% were lung cancers. After excluding hematological malignancies, 85/120 (70.83%) patients with cancer from the full pandemic period 26 February–30 December were diagnosed at Stage III–IV (Table 4).

## 4. Discussion

This is the first study to characterize the impact of COVID-19 on all diagnostic activity relevant to suspected cancer in patients referred from PC to a public hospital in Spain. We used data from prior pre-pandemic periods of 2020 and 2019 for comparison to address potential biases from seasonal and monthly variation. The study revealed huge changes through the diagnostic pathway. The number of people referred with concerning symptoms and the number subsequently diagnosed with cancer dropped sharply during the lockdown period and a decline persisted up until the end of 2020. COVID-19 had a deleterious impact on the access to key tests with significant delays through all pandemic periods. Delays in diagnosis and missing cancers because of such a gridlock imply longer times to enter treatment pathways, leaving disease progression unaffected.

The impact of changes on cancer brought about by the pandemic has been investigated throughout the full pathway from referral for suspected symptoms, diagnostic procedures, and number of people with new cancers referred for treatment [12,26,27]. The situation is explained by factors linked to both patients and the health system, as exemplified by people’s reluctance to seek medical care and disruptions on multiple services, respectively. This situation exists at a global scale and, although trends are similar in Spain, evidence is limited. One of few studies was a population-based survey that examined anticipated help seeking for cancer symptoms during the pandemic. Seeking help was consistently delayed with a 20–50% increase in the odds of waiting at least one week after symptom onset compared with a non-pandemic period. When asked about barriers to health seeking, participants were more likely to respond, “being worried about what the doctor may find” and “… about wasting the doctor’s time” [28].

The investigative management of symptomatic-but-as-yet-undiagnosed cancer can shape the interval between symptom onset and diagnosis. COVID-19 has had dire consequences on diagnostic service provision for cancer, as revealed in studies analyzing radiology and endoscopic practices. Data from a large healthcare system in the USA showed an average reduction in the weekly imaging volume of 28% during 1 March–18 April, 2020, compared with the same period in 2019. Volume reduction differed according to procedure types (e.g., 64% for ultrasound, 46% for CT, and 56% for interventional radiology [29]). Similar findings were reported in a global survey of nuclear medicine departments from 72 countries [30]. On average, the volume of nuclear medicine procedures for diagnosis during April 16–May 3 dropped by 54% (36% for PET/CT scans). While countries worldwide experienced a similar degree of decline, the impact on departments from countries that were in the post-spike phase of the pandemic when they responded to the survey was less pronounced [30].

Changes in endoscopic activity due to COVID-19 have also implied marked reductions in procedure volumes. Analyzing GI endoscopy reports from a UK national database, one study showed a substantial decline in the average weekly number of endoscopic procedures during 23 March–31 May, compared with a pre-pandemic period in 2020, with a 90% reduction for colonoscopy/flexible sigmoidoscopy, 86% for esophagogastroduodenoscopy, and 44% for ERCP [31]. Although activity improved by late May, it was only 20% of pre-COVID-19 activity. Additionally, comparing endoscopic diagnoses of cancer during the COVID-19 period with the number of expected cancers, missing cancers (i.e., esophageal, gastric, colorectal, and pancreatobiliary cancers) amounted to 58% [31]. Results were similar in a US study, though the average decline in new cancer diagnoses during the lockdown period was less pronounced [32]. Similar to the results from the above studies, we found a steep decline in weekly procedure volumes early in the pandemic, persisting in lower proportions for the rest of the year. An assessment of monthly numbers of mammograms and colonoscopies from PC healthcare registries in Catalonia showed a reduction of 66% and 65%, respectively, during lockdown, persisting for colonoscopies in a lower proportion (23%) in the post-lockdown period July–September 2020 [15]. To our knowledge, no study has described the impact on diagnostic procedures stratified by waiting times. Trends in waiting times were examined in this study across all modality types, revealing substantial increases during the COVID-impacted period before recovering partially for the rest of 2020. Importantly, cancer rates experienced a significant decline during the COVID-impacted period with a 54% reduction compared with the same 2019 period, persisting in smaller proportions afterwards.

This concerning decrease in the number of cancers is consistent across cancer sites. A cross-sectional study of patients across USA showed a 46% decline in the weekly number of six cancers combined (breast, colorectal, lung, pancreatic, gastric, and esophageal) during 1 March–18 April, with a partial recovery starting 29 March [33]. Additionally, using a national database where all cases of cancer were reported, a French article reported a 33% reduction in the average number of new cancers during lockdown (March–May) in 2020, which was consistent through all cancer sites including breast, colorectal, lung, pancreatic, and prostatic cancers. Despite a recovery trend after lockdown (June–September 2020), the number remained 19% lower than the average of 2018 and 2019 [34].

Slow and incomplete restoration trends in cancer and procedure volumes following the COVID-19 acme are consistent across published studies. In our institution, most of the original reasons for decreased activity including issues of people’s reluctance to present to PC doctors or attend hospital appointments and issues of staff relocation and readjustment of diagnostic services persisted all through the second wave period.

### 4.1. Limitations

Direct comparisons of our findings with existing literature are challenging because previous reports are based on population-based data. We focus on a population from an academic medical institution at the pandemic nucleus and national data or aggregation data from several QDUs were not used. From the start of lockdown to 30 December, the highest rates of cases and deaths in Spain were reported in Madrid (21% and 23%, respectively) and Catalonia (19% and 17%, respectively) communities [16]. Therefore, even if our results may serve as a worst-case scenario, analyses of imaging and endoscopic case volumes and waiting times in less severely affected regions would be warranted for comprehensive evaluation. Finally, treatment variables such as waiting times or treatment modalities in patients with a cancer diagnosis were not analyzed. The finding that 70.83% of epithelial cancers were diagnosed at Stage III–IV would however envisage fewer treatments with curative intent and worse survival rates in this population.

### 4.2. Implications

The international survey of nuclear medicine departments provided an overall picture of the factors heading up changes in diagnostic practices. The reduction in procedure volumes was attributed to a combination of incidents including shortages of personal protective equipment (50% of sites), SARS-CoV-2 infections (15% of staff (28% in Spain and Italy)), reduction of working hours (up to 26% of sites), staff redeployment to other departments (34%), and demand and supply disruptions [30]. Expertise gained from this, and similar cases, has been decisive to work out strategies and build resilience to confront the impact of new threats on healthcare services. While implications from our research are much like those addressed in the above studies, health providers and leaders may particularly learn through the UK management strategy to use the pandemic as an opportunity to advance the implementation of RDCs whilst building COVID-19-free diagnostic hubs/environments to address backlogs of delayed diagnosis and unmet needs [35].

## 5. Conclusions

This investigation has provided insight into the impact of COVID-19 on the diagnostic pathway of symptomatic-but-as-yet-undiagnosed cancer in Spain, from referral initiated by PC clinicians to a complete panel of investigative tests and procedures. Examining weekly trends from the start of lockdown throughout successive months and varying levels of restrictions, we have shown how time-critical access to cancer diagnostic services in patients with suspected symptoms collapsed. A multilevel failure effectively translated into substantial increases in waiting times for weekly procedures and reductions in procedure volumes and cancer diagnoses. Changes were more marked between March and April when Spanish hospitals experienced a massive surge in COVID-19 admissions. Even though a recovery started in the week commencing 30 April, when some restrictions were lifted, diagnostic activity and cancer rates did not return to baseline levels.

Exposing the burden of COVID-19 on diagnosis of suspected cancer can inform short-term and long-term practice decisions to protect healthcare cancer services and provide fast and efficient diagnostics ahead of new challenging periods.

## Figures and Tables

**Table 1:** Characteristics of patients referred to the Quick Diagnosis Unit during the COVID-impacted period and the same period in 2019.

	2019: 26 February–30 April	COVID-Impacted Period (2020: 26 February–28 April)	*p*-Value
**Referrals**			
Total, *n*	201	126	
**Age**, years, mean ± SD	68.22 ± 14.91	66.35 ± 13.73	0.1134
<65	70 (34.83)	47 (37.30)	
≥65	131 (65.17)	79 (62.70)	
**Sex**, *n* (%)			0.1324
Male	108 (53.73)	65 (51.59)	
Female	93 (46.27)	61 (48.41)	
**Household income per capita**, €, *n* (%) ^1^			0.1380
<18,000	105 (52.24)	63 (50.00)	
18,000–100,000	82 (40.80)	54 (42.86)	
>100,000	14 (6.97)	9 (7.14)	
**Comorbidity index**, *n* (%)			0.0556
0–1	141 (70.15)	94 (74.60)	
2	44 (21.89)	27 (21.43)	
≥3	16 (7.96)	5 (3.97)	
**Smoking status**, *n* (%)			0.1433
Current	64 (31.84)	37 (29.37)	
Former	48 (23.88)	30 (23.81)	
None	89 (44.28)	59 (46.83)	
**Alcohol intake**, *n* (%)			0.0786
Excessive	23 (11.44)	12 (9.52)	
Normal limits	49 (24.38)	26 (20.64)	
None	129 (64.18)	88 (69.84)	
**Presenting manifestations**, *n* (%)			
Unexplained weight loss/fatigue	31 (15.42)	22 (17.46)	
Fever of unknown origin/sweats	13 (6.48)	10 (7.94)	
Nonspecific gastrointestinal symptoms and signs	11 (5.47)	9 (7.14)	
Unexplained progressive pain (non-abdominal)	9 (4.48)	5 (3.97)	
Abdominal pain	14 (6.97)	10 (7.94)	
Effusions (ascites, pleural, pericardial)	9 (4.48)	4 (3.17)	
Jaundice/cholestasis	10 (4.98)	8 (6.35)	
Anemia	32 (15.92)	18 (14.29)	
Persistently abnormal laboratory tests	10 (4.98)	7 (5.56)	
Mass (abdominal/liver, lung/mediastinal, bone, brain)	14 (6.97)	10 (7.94)	
Lung consolidation/opacity	6 (2.99)	3 (2.38)	
Abnormal lymphadenopathy/lumps	16 (7.96)	8 (6.35)	
Overt blood loss (hematuria, hematochezia, hemoptysis, vaginal bleeding)	17 (8.46)	9 (7.14)	
Dyspnea/persistent cough	4 (1.99)	1 (0.79)	
Dysphagia	5 (2.49)	2 (1.59)	
**Diagnosis**, *n* (%)			
Benign organic diseases	137 (68.16)	84 (66.67)	
Cancer	43 (21.39)	20 (15.87)	
Other	21 (10.45)	22 (17.46)	
**Cancer: nature of presenting symptoms**, *n* (%)			0.1295
Focal	29 (67.44)	13 (65.00)	
Nonspecific	14 (32.56)	7 (35.00)	
**Cancer: pre-referral consultations**, *n* (%)			0.0092
1	22 (51.16)	9 (45.00)	
2	11 (25.58)	9 (45.00)	
≥3	10 (23.26)	2 (10.00)	
**Cancer: performance score**, *n* (%)			0.0337
0–1	20 (46.51)	11 (55.00)	
2	15 (34.88)	6 (30.00)	
3–4	8 (18.60)	3 (15.00)	
**Nonmalignant organic diseases**, *n* (%)			
Hematological	4 (2.92)	1 (1.19)	
Gastrointestinal diseases	33 (24.09)	19 (22.62)	
Genitourinary	6 (4.38)	3 (3.57)	
Respiratory	4 (2.92)	4 (4.76)	
Infections (viral)	7 (5.11)	5 (5.95)	
Gynecological	6 (4.38)	2 (2.38)	
Bone	7 (5.11)	4 (4.76)	
Hepatobiliary/pancreatic diseases	15 (10.95)	10 (11.90)	
Benign neoplasms/reactive lymphadenitis	6 (4.38)	4 (4.76)	
Rheumatic/autoimmune/granulomatous diseases	9 (6.57)	5 (5.95)	
Endocrine diseases	7 (5.11)	4 (4.76)	
Other	33 (24.09)	23 (27.38)	

^1^ According to the Catalan Health Surveillance System (CHSS) database (Health Department. Government of Catalonia).

**Table 2:** Referrals, appointments, and procedure volumes across study periods in 2020.

	Period (2020)				
	Pre-COVID	COVID-Impacted	De-Escalation	Second Wave	26 February–30 December
	1 January–25 February	26 February–28 April	29 April–30 June	1 July–30 December	
**Referrals**					
Total, n	176	126	169	504	799
Mean (weekly) ± SD	22.00 ± 3.38	14.00 ± 3.46	18.78 ± 2.11	19.38 ± 2.08	18.16 ± 3.19
*p*-value	0.6301	<0.0001	0.0149	0.0009	<0.0001
**Appointments per case**, mean ± SD	3.13 ± 0.35	2.89 ± 0.60	3.00 ± 0.50	3.23 ± 0.43	3.11 ± 0.49
*p*-value	>0.9999	0.4442	0.6755	0.5380	0.8321
**Ratio of successive/first visits**, mean ± SD	1.98 ± 0.23	1.77 ± 0.54	1.90 ± 0.48	2.08 ± 0.37	1.98 ± 0.44
**Ratio of telehealth/total visits**, %	0.37	58.51	30.27	17.67	33.56
**Diagnostic procedure**					
**GI endoscopy/USE**					
Number per period (vs. 2019)	68 (vs. 69)	43 (vs. 76)	58 (vs. 77)	184 (vs. 227)	285 (vs. 380)
Mean ± SD	8.50 ± 1.41	4.78 ± 2.17	6.44 ± 1.74	7.08 ± 1.81	6.48 ± 2.04
Percent reduction (95% CI)	−3.17 (−21.97–15.62)	42.04 (22.66–61.42)	24.18 (14.26–34.09)	14.24 (5.73–22.75)	21.96 (14.97–28.95)
*p*-value	0.9022	0.0058	0.0324	0.0187	<0.0001
**CT scan**					
Number per period (vs. 2019)	57 (vs. 59)	34 (vs. 63)	50 (vs. 64)	152 (vs. 179)	236 (vs. 306)
Mean ± SD	7.13 ± 1.55	3.78 ± 1.20	5.56 ± 1.13	5.85 ± 1.41	5.36 ± 1.53
Percent reduction (95% CI)	1.19 (−14.96–17.34)	39.42 (17.75–61.08)	19.66 (7.58–31.73)	10.53 (2.65–18.41)	18.30 (11.20–25.40)
*p*-value	0.7682	0.0009	0.0509	0.0502	0.0001
**PET/CT scan**					
Number per period (vs. 2019)	24 (vs. 26)	16 (vs. 27)	23 (vs. 30)	69 (vs. 82)	108 (vs. 139)
Mean ± SD	3.00 ± 1.51	1.78 ± 1.09	2.56 ± 1.13	2.65 ± 1.33	2.46 ± 1.27
Percent reduction (95% CI)	−4.79 (−57.46–47.88)	34.45 (6.70–62.19)	15.56 (−13.77–44.88)	9.36 (−4.32–23.04)	15.76 (4.89–26.63)
*p*-value	0.7166	0.0746	0.2474	0.2111	0.0199
**Ultrasonography**					
Number per period (vs. 2019)	45 (vs. 47)	32 (vs. 50)	40 (vs. 52)	122 (vs. 142)	194 (vs. 244)
Mean ± SD	5.63 ± 2.39	3.56 ± 2.19	4.44 ± 1.51	4.69 ± 1.69	4.41 ± 1.78
Percent reduction (95% CI)	5.00 (−7.99–17.99)	38.37 (17.36–59.39)	22.72 (6.00–39.45)	13.60 (3.23–23.96)	20.53 (12.45–28.61)
*p*-value	0.8381	0.0412	0.0694	0.1132	0.0024
**Invasive procedures**					
Number per period (vs. 2019)	19 (vs. 20)	11 (vs. 22)	13 (vs. 21)	46 (vs. 60)	70 (vs. 103)
Mean ± SD	2.38 ± 1.19	1.22 ± 0.97	1.44 ± 0.53	1.77 ± 0.95	1.59 ± 0.90
Percent reduction (95% CI)	2.50 (−25.00–30.00)	47.22 (17.87–76.58)	29.63 (6.73–52.53)	17.37 (5.87–28.87)	25.99 (16.13–35.84)
*p*-value	0.8368	0.0189	0.0314	0.0729	0.0006
**Biopsy/cytology procedures**					
(ambulatory)					
Number per period (vs. 2019)	21 (vs. 23)	13 (vs. 25)	19 (vs. 26)	59 (vs. 72)	91 (vs. 123)
Mean ± SD	2.63 ± 1.06	1.44 ± 0.88	2.11 ± 0.60	2.27 ± 1.00	2.07 ± 0.95
Percent reduction (95% CI)	5.21(−19.75–30.17)	40.37 (12.00–68.74)	25.00 (8.05–41.95)	15.38 (1.70–29.07)	22.46 (12.33–32.59)
*p*-value	0.6337	0.0116	0.0144	0.0596	0.0003

Abbreviations: GI endoscopy/USE, gastrointestinal endoscopy/endoscopic ultrasonography; CT, computed tomography; PET/CTscan, positron emission tomography integrated with CT scan; CI, confidence interval.

**Table 3:** Waiting times across study periods in 2020 and percent differences compared with 2019.

Period (2020)	Waiting Times (Days, Mean ± SD)	Percent Increase (95% CI)	*p*-Value
	**Appointments**		
Pre-COVID (1 January–25 February)	2.59 ± 0.21	−1.78 (−11.73–8.17)	0.5911
COVID-impacted (26 February–28 April)	5.61 ± 0.61	106.80 (87.46–126.20)	<0.0001
De-escalation (29 April–30 June)	4.59 ± 0.24	75.41 (59.54–91.29)	<0.0001
Second wave (July 1–30 December)	3.80 ± 0.38	48.14 (39.83–56.45)	<0.0001
26 February–30 December	4.33 ± 0.83	65.72 (56.09–75.36)	<0.0001
	**GI endoscopy/USE**		
Pre-COVID (1 January–25 February)	5.45 ± 0.44	−0.54 (−7.84–6.76)	0.8305
COVID-impacted (26 February–28 April)	14.32 ± 3.24	161.50 (111.60–211.40)	<0.0001
De-escalation (29 April–30 June)	10.45 ± 0.84	85.58 (66.33–104.80)	<0.0001
Second wave (1 July–30 December)	8.67 ± 0.69	58.11 (51.74–64.49)	<0.0001
26 February–30 December	10.19 ± 2.71	84.88 (69.06–100.70)	<0.0001
	**CT scan**		
Pre-COVID (1 January–25 February)	4.22 ± 0.29	−0.09 (−8.18–8.01)	0.8880
COVID-impacted (26 February–28 April)	11.46 ± 2.62	171.20 (118.20–224.10)	<0.0001
De-escalation (29 April–30 June)	8.11 ± 0.96	90.61 (69.35–111.90)	<0.0001
Second wave (1 July–30 December)	6.87 ± 0.59	62.33 (54.73–69.93)	<0.0001
26 February–30 December	8.06 ± 2.22	90.37 (73.52–107.20)	<0.0001
	**PET** **/CT scan**		
Pre-COVID (1 January–25 February)	4.17 ± 0.54	0.74 (−11.88–13.35)	0.9880
COVID-impacted (26 February–28 April)	10.36 ± 2.28	152.50 (98.21–206.90)	<0.0001
De-escalation (29 April–30 June)	7.26 ± 1.31	74.60 (47.82–101.40)	<0.0001
Second wave (1 July–30 December)	6.47 ± 0.60	55.84 (48.52–63.16)	<0.0001
26 February–30 December	7.43 ± 1.96	79.45 (63.35–95.56)	<0.0001
	**Ultrasonography**		
Pre-COVID (1 January–25 February)	3.35 ± 0.37	−1.44 (−10.11–7.22)	0.7302
COVID-impacted (26 February–28 April)	8.39 ± 2.39	148.40 (89.28–207.50)	<0.0001
De-escalation (29 April–30 June)	6.20 ± 0.61	85.69 (65.26–106.10)	<0.0001
Second wave (1 July–30 December)	5.35 ± 0.51	58.95 (49.97–67.93)	<0.0001
26 February–30 December	6.15 ± 1.65	82.72 (66.73–98.71)	<0.0001
	**Invasive procedures**		
Pre-COVID (1 January–25 February)	5.16 ± 0.47	−0.70 (−8.78–7.38)	0.8175
COVID-impacted (26 February–28 April)	15.53 ± 3.81	200.70 (144.20–257.20)	<0.0001
De-escalation (29 April–30 June)	11.48 ± 1.29	122.90 (95.82–150.00)	<0.0001
Second wave (1 July–30 December)	8.80 ± 0.64	70.64 (65.46–75.82)	<0.0001
26 February–30 December	10.73 ± 3.23	107.90 (88.66–127.20)	<0.0001
	**Biopsy/cytology procedures**		
Pre-COVID (1 January–25 February)	3.82 ± 0.36	−0.91 (−6.77–4.94)	0.8230
COVID-impacted (26 February–28 April)	8.16 ± 2.22	111.20 (71.97–150.40)	<0.0001
De-escalation (29 April–30 June)	6.63 ± 0.52	73.19 (64.29–82.09)	<0.0001
Second wave (1 July–30 December)	5.84 ± 0.36	52.68 (47.17–58.19)	<0.0001
26 February–30 December	6.48 ± 1.37	68.85 (58.51–79.18)	<0.0001
	**Time-to-diagnosis**		
Pre-COVID (1 January–25 February)	10.38 ± 0.59	0.84 (−8.22–9.89)	0.9258
COVID-impacted (26 February–28 April)	20.14 ± 3.72	97.04 (65.15–128.90)	<0.0001
De-escalation (29 April–30 June)	15.54 ± 0.90	51.17 (41.99–60.35)	<0.0001
Second wave (1 July–30 December)	13.64 ± 0.86	32.87 (28.47–37.27)	<0.0001
26 February–30 December	15.36 ± 3.12	49.74 (39.85–59.62)	<0.0001

Abbreviations: See Table 2.

**Table 4:** Cancer sites and stages across study periods in 2020.

	Period (2020)				
	Pre-COVID	COVID-Impacted	De-Escalation	Second Wave	26 February–30 December
Total, *n* (%)	38/176 (21.59)	20/126 (15.87)	29/169 (17.16)	93/504 (18.45)	142/799 (17.77)
Mean (weekly) ± SD	4.75 ± 1.28	2.22 ± 1.48	3.22 ± 1.72	3.58 ± 1.30	3.23 ± 1.49
Percent reduction (95% CI)	−2.59 (−24.09–18.92)	54.07 (32.45–75.70)	30.03 (−1.89–61.94)	17.55 (2.63–32.47)	27.57 (15.91–39.23)
*p*-value	0.8631	0.0005	0.0439	0.0028	<0.0001
Primary site/stage III-IV, *n* (%)/(%)					
Pancreatic	7 (18.42)/5 (71.43)	4 (20.00)/3 (75.00)	6 (20.70)/4 (66.67)	15 (16.13)/12 (80.00)	25 (17.61)/19 (76.00)
Colorectal	6 (15.79)/3 (50.00)	3 (15.00)/1 (33.33)	5 (17.24)/2 (40.00)	18 (19.35)/10 (55.56)	26 (18.31)/13 (50.00)
Hematological	6 (15.79)/(n.a.)	3 (15.00)/(n.a.)	4 (13.79)/(n.a.)	15 (16.13)/(n.a.)	22 (15.49)/(n.a.)
Lung	5 (13.16)/3 (60.00)	2 (10.00)/1 (50.00)	4 (13.79)/3 (75.00)	13 (13.98)/11 (84.62)	19 (13.38)/15 (78.95)
Upper gastrointestinal tract	3 (7.89)/2 (66.67)	1 (5.00)/1 (100.00)	2 (6.90)/1 (50.00)	6 (6.45)/6 (100.00)	9 (6.34)/8 (88.89)
Renal and bladder	2 (5.26)/1 (50.00)	1 (5.00)/0 (0.00)	2 (6.90)/1 (50.00)	4 (4.30)/1 (25.00)	7 (4.93)/2 (28.57)
Hepatobiliary	2 (5.26)/2 (100.00)	1 (5.00)/1 (100.00)	1 (3.45)/1 (100.00)	4 (4.30)/3 (75.00)	6 (4.23)5 (83.33)
Breast	2 (5.26)/1 (50.00)	2 (10.00)/2 (100.00)	2 (6.90)/2 (100.00)	7 (7.53)/5 (71.43)	11 (7.75)/9 (81.82)
Prostate	2 (5.26)/2 (100.00)	1 (5.00)/1 (100.00)	1 (3.45)/1 (100.00)	3 (3.23)/2 (66.67)	5 (3.52)/4 (80.00)
Gynecological ^1^	1 (2.63)/0 (0.00)	1 (5.00)/1 (100.00)	1 (3.45)/1 (100.00)	4 (4.30)/3 (75.00)	6 (4.23)/5 (83.33)
Cancer of unknown primary site	1 (2.63)/1 (100.00)	0 (0.00)/(n.a.)	0 (0.00)/(n.a.)	1 (1.08)/1 (100.00)	1 (0.70)/1 (100.00)
Sarcoma	1 (2.63)/1 (100.00)	0 (0.00)/(n.a.)	0 (0.00)/(n.a.)	2 (2.15)/1 (50.00)	2 (1.41)/1 (50.00)
Neuroendocrine tumor	0 (0.00)/(n.a.)	0 (0.00)/(n.a.)	1 (3.45)/1 (100.00)	0 (0.00)/(n.a.)	1 (0.70)/1 (100.00)
Malignant melanoma	0 (0.00)/(n.a.)	1 (5.00)/1 (100.00)	0 (0.00)/(n.a.)	0 (0.00)/(n.a.)	1 (0.70)/1 (100.00)
Mesothelioma	0 (0.00)/(n.a.)	0 (0.00)/(n.a.)	0 (0.00)/(n.a.)	1 (1.08)/1 (100.00)	1 (0.70)1 (100.00)
Stage III-IV ^2^, total *n* (%)	21/32 (65.63)	12/17 (70.59)	17/25 (68.00)	56/78 (71.79)	85/120 (70.83)

^1^ Ovarian, endometrial, and cervical cancer; ^2^ Excluding hematological malignancies.

## Data Availability

The data that support the findings of this study are available from the corresponding author on reasonable request.

## Supplementary Materials

The following are available online at https://www.mdpi.com/article/10.3390/diagnostics11112096/s1, Table S1: Referral criteria of Quick Diagnosis Unit; Table S2: Study characteristics in pre-pandemic periods of 2020 and 2019; Table S3: Procedure volumes and waiting times across study periods in 2020.
