# Supplementary material for: Delays in Cancer Diagnostic Testing at a Quick Referral Unit in Spain during COVID-19
Source: Diagnostics (Basel). 2021 Nov 12;11(11):2096. doi: 10.3390/diagnostics11112096 (PMC8623804; doi:10.3390/diagnostics11112096)
Supplement: Supplementary file 1 [file diagnostics-11-02096-s001.zip › diagnostics-1397420-supplementary.pdf]

Table S1. Referral criteria of Quick Diagnosis Unit

**Unintentional weight loss/severe fatigue**
**Persistent nausea/appetite loss**
**Fever of unknown origin**
**Anemia**
**Change in bowel habit**
**Abdominal pain**
**Rectal bleeding**
**Dysphagia**
**Palpable abdominal mass**
**Hepatomegaly/liver mass**
**Jaundice**
**Exudative ascites**
**Dyspnea**
**Persistent cough**
**Hemoptysis**
**Lymphadenopathy or palpable lumps**
**Hematuria**
**Metrorrhagia**
**Suspected tumor in imaging investigation**
**Pain (other than abdominal)**

Table S2. Study characteristics of patients from pre-pandemic periods of 2020 and 2019.

	Pre-Covid 2019 (Jan 1–Feb 26)	Pre-Covid 2020 (Jan 1–Feb 26)	*P*-value
**Referrals**			
**Total, n**	183	176	
**Mean (weekly) ± SD**	22.88 ± 3.72	22.00 ± 3.38	0.6301
**Appointments per case, mean ± SD**	3.13 ± 0.64	3.13 ± 0.35	>0.9999
**Procedures, mean ± SD**			
**GI endoscopy/USE**	8.63 ± 2.45	8.50 ± 1.41	0.9022
**CT scan**	7.38 ± 1.77	7.13 ± 1.55	0.7682
**PET/CT scan**	3.25 ± 1.17	3.00 ± 1.51	0.7166
**Ultrasonography**	5.88 ± 2.42	5.63 ± 2.39	0.8381
**Invasive procedures**	2.50 ± 1.20	2.38 ± 1.19	0.8368
**Biopsy/cytology procedures (ambulatory)**	2.88 ± 0.99	2.63 ± 1.06	0.6337
**Waiting times, days, mean ± SD**			
**Appointment time**	2.66 ± 0.31	2.59 ± 0.21	0.5911
**Time to GI endoscopy/USE**	5.50 ± 0.45	5.45 ± 0.44	0.8305
**Time to CT scan**	4.24 ± 0.30	4.22 ± 0.29	0.8880
**Time to PET/CT scan**	4.17 ± 0.43	4.17 ± 0.54	0.9880
**Time to ultrasonography**	3.40 ± 0.26	3.35 ± 0.37	0.7302
**Time to invasive procedures**	5.20 ± 0.33	5.16 ± 0.47	0.8175
**Time to biopsy/cytology procedures**	3.86 ± 0.34	3.82 ± 0.36	0.8230
**Time-to-diagnosis**	10.35 ± 0.67	10.38 ± 0.59	0.9258
**Age, years, mean ± SD**	69.75 ± 13.28	68.20 ± 12.15	0.2142
**Males, n (%)**	97 (53.01)	91 (51.70)	0.2236
**Comorbidity index, n (%)**			0.1185
**0-1**	124 (67.76)	124 (70.45)	
**2**	43 (23.50)	39 (22.16)	
**>3**	16 (8.74)	13 (7.39)	
**Presenting manifestations, n (%)**			
**Unexplained weight loss/fatigue**	27 (14.75)	28 (15.91)	
**Fever of unknown origin/sweats**	12 (6.56)	13 (7.39)	
**Abdominal pain**	12 (6.56)	14 (7.95)	
**Anemia**	26 (14.21)	27 (15.34)	
**Mass (abdominal/liver, lung/mediastinal, bone, brain)**	14 (7.65)	11 (6.25)	
**Overt blood loss**	15 (8.20)	12 (6.82)	
**Dyspnea/persistent cough**	4 (2.19)	3 (1.70)	
**Dysphagia**	2 (1.09)	2 (1.14)	
**Other**	71 (38.80)	66 (37.50)	
**Diagnosis, n (%)**			
**Benign organic diseases**	128 (69.95)	118 (67.05)	
**Cancer**	39 (21.31)	38 (21.59)	
**Other**	16 (8.74)	20 (11.36)	
**Benign organic diseases, n (%)**	128	118	
**Gastrointestinal diseases**	32 (25.00)	28 (23.73)	
**Hepatobiliary/pancreatic diseases**	15 (11.72)	12 (10.17)	
**Rheumatic/autoimmune/granulomatous diseases**	7 (5.47)	9 (7.63)	
**Other**	74 (57.81)	69 (58.47)	
**Cancer: nature of presenting symptoms, n (%)**			0.3247
**Focal**	27 (69.23)	26 (68.42)	
**Nonspecific**	12 (30.77)	12 (31.58)	
**Cancer: pre-referral consultations, n (%)**			0.0955
**1**	21 (53.85)	19 (50.00)	
**2**	10 (25.64)	10 (26.32)	
**>3**	8 (20.51)	9 (23.68)	
**Cancer: performance score, n (%)**			0.0943
**0-1**	17 (43.59)	18 (47.37)	
**2**	14 (35.90)	13 (34.21)	

**3-4**	8 (20.51)	7 (18.42)
**Cancer: primary site/stage III-IV, n (%)/(%)**		
**Pancreatic**	7 (17.95)/6 (85.71)	7 (18.42)/5 (71.43)
**Colorectal**	6 (15.38)/3 (50.00)	6 (15.79)/3 (50.00)
**Hematological**	5 (12.82)/(n.a.)	6 (15.79)/(n.a.)
**Lung**	6 (15.38)/4 (66.67)	5 (13.16)/3 (60.00)
**Upper GI tract**	3 (7.69)/2 (66.67)	3 (7.89)/2 (66.67)
**Renal and bladder**	3 (7.69)/2 (66.67)	2 (5.26)/1 (50.00)
**Hepatobiliary**	2 (5.13)/2 (100.00)	2 (5.26)/2 (100.00)
**Breast**	2 (5.13)/1 (50.00)	2 (5.26)/1 (50.00)
**Prostate**	1 (2.56)/1 (100.00)	2 (5.26)/2 (100.00)
**Gynecological**	1 (2.56)/1 (100.00)	1 (2.63)/0 (0.00)
**Other**	3 (7.69)/2 (66.67)	2 (5.26)/2 (100.00)
**Stage III-IV, total n (%)** [1]	24/34 (70.59)	21/32 (65.63)

[1] Excluding hematological malignancies.

Table S3. Procedure volumes and waiting times across study periods in 2020.

	Period (2020)			
	**Pre-Covid**	**Covid-impacted**	**De-ecalation**	**Second wave**
**Bronchoscopy/endobronchial ultrasonography**				
**Number, mean ± SD**	0.88 ± 0.83	0.44 ± 0.53	0.56 ± 0.53	0.65 ± 056
**Waiting time, days, mean ± SD**	5.12 ± 0.42	13.90 ± 3.27	10.52 ± 0.95	9.16 ± 0.59
**Magnetic resonance image**				
**Number, mean ± SD**	1.63 ± 0.74	1.11 ± 0.78	1.33 ± 0.87	1.46 ± 0.65
**Waiting time, days, mean ± SD**	4.60 ± 0.38	11.18 ± 2.31	9.35 ± 1.07	8.27 ± 0.60
**Bone scintigraphy**				
**Number, mean ± SD**	0.88 ± 0.64	0.33 ± 0.50	0.56 ± 0.53	0.62 ± 0.50
**Waiting time, days, mean ± SD**	3.47 ± 0.28	6.57 ± 2.12	5.44 ± 0.82	4.34 ± 0.63
**Bone marrow biopsy/aspiration**				
**Number, mean ± SD**	0.75 ± 0.46	0.22 ± 0.44	0.33 ± 0.50	0.50 ± 0.51
**Waiting time, days, mean ± SD**	3.67 ± 0.33	6.51 ± 2.05	5.24 ± 0.74	4.08 ± 0.47
